# Nutritional profiling of hilsa (*Tenualosa ilisha*) of different size groups and sensory evaluation of their adults from different riverine systems

**DOI:** 10.1038/s41598-019-55845-w

**Published:** 2019-12-17

**Authors:** D. De, S. Mukherjee, P. S. Shyne Anand, P. Kumar, V. R. Suresh, K. K. Vijayan

**Affiliations:** 1 Kakdwip Research Centre of ICAR-Central Institute of Brackishwater Aquaculture, Kakdwip, South 24 Parganas, West Bengal 743 347 India; 2 ICAR-Central Institute of Brackishwater Aquaculture, 75, Santhome High Road, Chennai, Tamil Nadu 600028 India; 3 ICAR-Central Inland Fisheries Research Institute, Barrackpore, West Bengal 700 120 India; 4 Present Address: Principal Scientist, ICAR-Central Institute of Brackishwater Aquaculture, 75, Santhome High Road, Chennai, Tamil Nadu 600 028 India

**Keywords:** Biochemistry, Ichthyology

## Abstract

Nutritional composition of hilsa, *Tenualosa ilisha*, of different size groups was analyzed to study variations in their composition with the progression of growth, and to correlate it with the flavor of adult hilsa (>800 g size) collected from different riverine systems (Hooghly and Padma). The amino acid analysis revealed significantly higher arginine (P < 0.01), methionine (P < 0.01) and glycine (P < 0.05) contents in samples below 5 g, whereas samples above 800 g had higher (P < 0.01) leucine and isoleucine contents. Total saturated and monounsaturated fatty acids were lower (P < 0.01) in fish below 5 g as compared to larger size groups (>5 g), whereas docosahexaenoic acid was higher (P < 0.01) in fish below 5 g size. Nutritional composition of adult hilsa (>800 g) from Hooghly and Padma river revealed higher (P < 0.01) aspartic acid, glutamic acid, alanine, palmitoleic and oleic acid in samples from the Padma, whereas leucine and isoleucine contents were higher (P < 0.01) in hilsa from Hooghly. Sensory evaluation test revealed superior (P < 0.05) taste, aroma, and muscle texture of hilsa from the Padma as compared to those from Hooghly. Higher alanine, aspartic acid, glutamic acid, oleic acid, and palmitoleic acid along with higher n3:n6 fatty acid are attributed to the superior taste of hilsa from the Padma.

## Introduction

Hilsa, *Tenualosa ilisha* belonging to the class Actinopterygii, order Clupeiformes, sub-family Alosinea, and family Clupeidae is one of the most important fishes of the Indo-Pacific region. The hilsa shad occurs in the foreshore areas, estuaries, brackish water lakes and freshwater rivers of the western division of the Indo-Pacific faunistic region^1^. This species is particularly high in abundance in the Indian State of West Bengal and Bangladesh waters, mainly along the river Hooghly, the Indian limb of river Ganga and its coastal areas and in river Padma, the main limb of river Ganga passing through Bangladesh. Due to its taste, flavor and culinary properties, the fish has high demand, and economic value in West Bengal and Bangladesh^2^. Bengali population in India have a perception that hilsa from the Padma river is tastier than hilsa from Hooghly, which lead to high demand for Padma hilsa in India. However, there is a scarcity of information on the reason behind the difference in flavor and taste of the fish from the Hooghly and Padma river systems.

Fish is a food of excellent nutritional value, providing high-quality protein and a wide variety of vitamins and minerals, including vitamins A and D, phosphorus, magnesium, selenium, iodine, etc.^3^. Fish protein is easily digestible and contains a high amount of lysine and other sulfur-containing amino acids like methionine and cysteine and favorably complements dietary protein from plant sources^4^. Fish or fish oil contains omega-3 polyunsaturated fatty acids (PUFAs), e.g., docosahexaenoic acid (DHA) and eicosapentaenoic acid (EPA), which are beneficial for human health and reduce the risk of coronary heart diseases^5^. Earlier works on the nutritional composition of hilsa were basically on larger size groups focusing on its benefits for human health. However, no systematic information is available in the case of different size groups. Attempts for captive breeding and pond based culture as well as the formulation of artificial feed for hilsa has gained momentum in India. Knowledge on the biochemical composition of fish of different size groups is important to understand the critical nutrient requirement for different life stages of growth and reproduction of the fish under captive condition^6,7^. Hence the nutritional composition of different size groups of the fish (below 5 g to above 800 g) collected from different habitats, viz., marine, brackish water and freshwater were investigated to have an understanding on changes of body nutritional composition with the progression of growth which will help in development of feed for hilsa of different stages. Simultaneously, a comparative study on sensory evaluation and nutritional composition of adult hilsa from Hooghly and Padma rivers were also taken up to investigate the reason behind the difference in taste and flavor between Hooghly and Padma hilsa.

## Materials and Methods

### Sample collection

**Figure 1:**
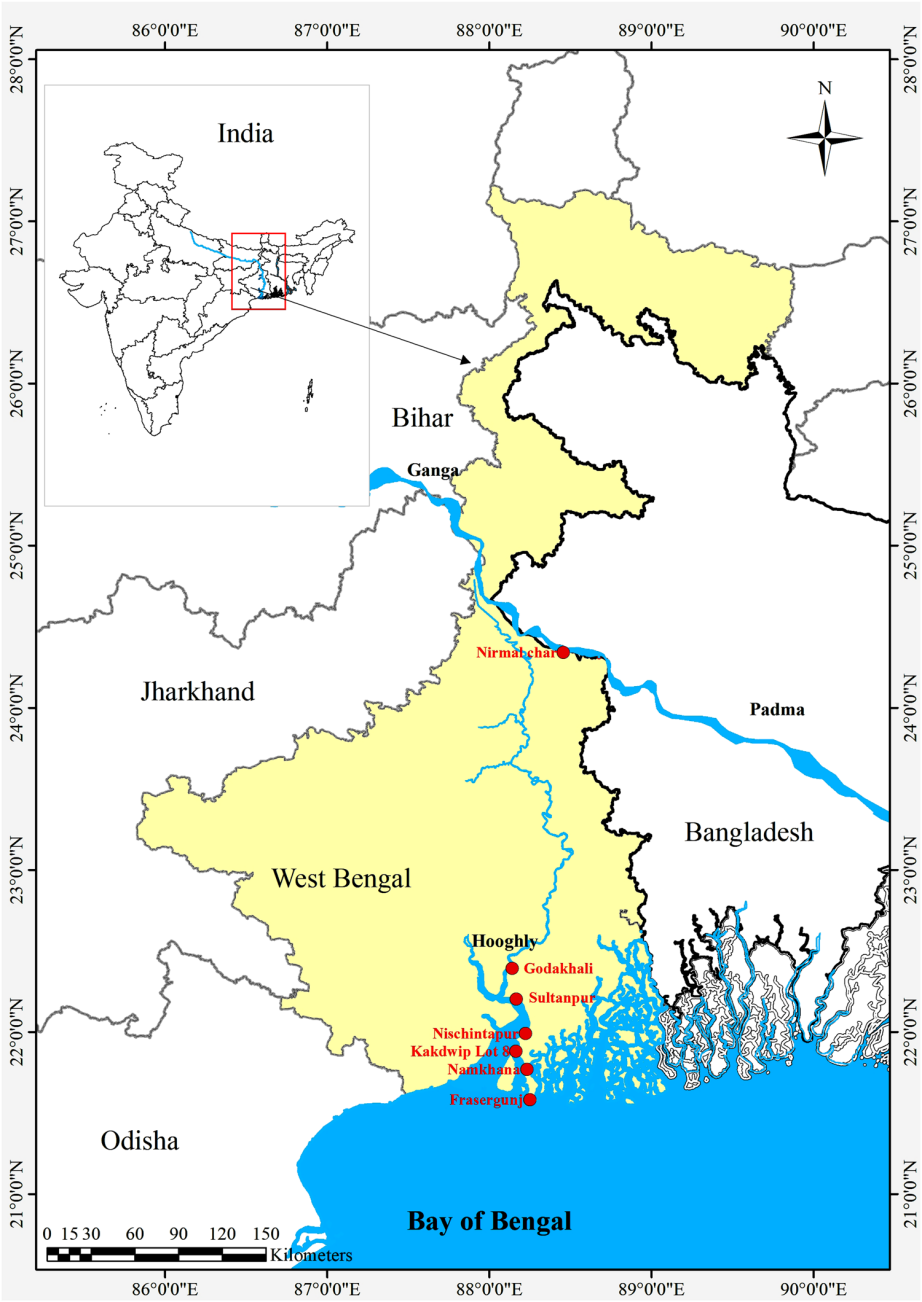
Map of sample collection sites (marked in red color) in Hooghly river and Padma river systems.

Specimens of *T. ilisha* (n = 350 with 50 each in 7 size groups) ranging from 0.4 g to 900 g were collected from different habitats/sampling sites (Fig. 1) covering marine (Namkhana and Frasergunj), Brackish water zone (Kakdwip lot 8, Nischintapur, Sultanpur) and freshwater (Godakhali) of Hooghly riverine system in West Bengal, India (Table 1). Necessary permission was obtained from Directorate of Fisheries, Government of West Bengal, India for collecting hilsa of different size groups from different habitats. The samples were divided into seven different size groups according to their body weight as <5 g (group I), 5–100 g (group II), 101–200 g (group III), 201–400 g (group IV), 401–600 g (group V), 601–800 g (group VI) and >800 g (group VII) to study the variation in nutritional composition with the advancement of growth. Hilsa specimens (n = 50) of >800 g (group VIII) were also collected from Padma river (Nirmal char) during December. A comparative study on the nutritional composition and sensory evaluation was made to determine the reason behind the difference in taste and flavor, if any, between adult hilsa (weight >800 g and length 390 to 418 mm size) from the Hooghly and Padma river systems collected during December (Fig. 1). Fish samples were collected while local fishers were fishing them in the respective stations. Immediately after collection, the samples were transported to the laboratory in chilled condition. Once in the laboratory, using sterile scissors and knife, the head portions of the samples were removed, and the rest of the body was minced after pooling the samples of the same size group. For large size fish, after removal of head, steaks (which is cut perpendicular to the spine) were made from each sample, and two representative steaks from each portion (anterior, middle and posterior) of each fish were taken and pooled group wise irrespective of the site of collection and minced for different biochemical analysis.

**Table 1:** Details of collection site of different groups of *Tenualosa ilisha*.

Size group of fish (g)	Group	Source of the collection (Habitat)	Latitude (N)	Longitude (E)	Salinity (ppt)	No. of samples	Plankton status in gut	Collection Season (2013–2016)
<5	I^a^	Kakdwip lot 8 (Brackishwater)	21.8820°	88.1645°	3–15	50	*Coscinodiscus* sp.*, Biddulphia* sp.	January- March, May, June, August- December
5–100	II^a^	Frasergunj (Marine)	21.5560°	88.2263°	30–35	20	*Coscinodiscus* sp.*, Biddulphia* sp., Copepod*, Nitzschia* sp., *Pleurosigma* sp., *Thalassiothrix* sp.	September, November, December
		Namkhana (Marine)	21.7699°	88.2315°	20–30	15		
		Kakdwip lot 8 (Brackishwater)	21.8820°	88.1645°	2–13	15		
101–200	III^a^	Frasergunj (Marine)	21.5560°	88.2263°	30–35	18	*Coscinodiscus* sp.*, Biddulphia* sp., Copepod*, Nitzschia* sp., *Pleurosigma* sp., *Thalassiothrix* sp., *Ulothrix* sp., *Ceratium* sp.	March, May, August, December
		Namkhana (Marine)	21.7699°	88.2315°	20–30	19		
		Kakdwip lot 8 (Brackishwater)	21.8820°	88.1645°	2–13	13		
201–400	IV^a^	Godakhali (Freshwater)	22.3923°	88.2023°	0	42	Copepod, *Coscinodiscus* sp., *Biddulphia* sp.*, Ulothrix* sp.*, Chaetomorpha* sp.*, Diatoma* sp., *Coccolithophore, Asterionella* sp.*, Diploneis* sp.*, Oscillatoria* sp.	March, May, August, September
		Kakdwip lot 8 (Brackishwater)	21.8820°	88.1645°	2–13	8		
401–600	V^a^	Frasergunj (Marine)	21.556°	88.2263°	30–35	11	*Biddulphia* sp.,Copepod, *Ulothrix* sp., *Chaetomorpha* sp., *Diatoma* sp., *Nitzschia* sp., *Pleurosigma* sp., *Asterionella* sp.,	March, June, August, November
		Namkhana (Marine)	21.7699°	88.2315°	20–30	9		
		Kakdwip lot 8 (Brackishwater)	21.8820°	88.1645°	2–13	5		
		Godakhali (Freshwater)	22.3923^o^	88.2023°	0	25		
601–800	VI^a^	Frasergunj (Marine)	21.5560°	88.2263°	30–35	13	*Biddulphia* sp.,Copepod, *Ulothrix* sp., *Chaetomorpha* sp., *Diatoma* sp., *Nitzschia* sp., *Pleurosigma* sp., *Asterionella* sp.*, Diploneis* sp., *Rhizosolenia* sp.*, Coscinodiscus* sp.	March, August, September, November, December
		Namkhana, (Marine)	21.7699°	88.2315°	20–30	11		
		Kakdwip (Brackishwater)	21.8820°	88.1645°	2–13	12		
		Sultanpur (Brackishwater)	22.1987°	88.2023°	0.5–3	9		
		Godakhali (Freshwater)	22.3923°	88.2023°	0	5		
>800	VII^a^	Kakdwip lot 8 (Brackishwater)	21.8820°	88.1645°	2–13	34	*Coscinodiscus* sp., *Biddulphia* sp., *Nitzschia* sp., *Diatoma* sp., Copepod	December
		Nischintapur (Brackishwater)	21.9978°	88.1908°	2–13	16		
>800	VIII^b^	Nirmal char (Freshwater, Padma river)	24.340205°	88.4560°	0	50	Copepod, *Cladocerans, Ulothrix* sp.	December

^a^Fish of groups I to VII were collected from different locations of Hooghly river system.

^b^Fish of group VIII were collected from the Padma river system.

All specimen handling and analysis were done in accordance with relevant guidelines and regulations for dealing with live animal and approved by the Institutional Animal Ethics committee of ICAR-Central Institute of Brackishwater Aquaculture, Chennai, India.

### Biochemical analysis

Proximate analysis was done following official methods of analysis of AOAC International^8^. The moisture and dry matter contents were determined using a hot air oven by drying the sample at 105 °C for 8 hours. Ash content was determined at 600 °C. Nitrogen content was estimated by Kjeldahl method (Kelplus Classic, DX VA, Pelican Equipment) and crude protein was calculated by multiplying nitrogen percentage by 6.25. Lipid was determined by the solvent extraction method by Soxtec system (SOCS Plus, SCS-6, Pelican Equipment) using diethyl ether (boiling point, 40–60 °C) as a solvent. The crude fiber was determined by digesting the moisture and fat-free sample successively with dilute (1.25%) acid and alkali using Fibre cap (Foss tecator, Sweden). Nitrogen free extract (NFE) was determined as per the following formula

$${\rm{NFE}}\,( \% )=100\,\mbox{--}\,( \% \,{\rm{moisture}}+ \% \,{\rm{crude}}\,{\rm{protein}}+ \% \,{\rm{crude}}\,{\rm{lipid}}+ \% \,{\rm{crude}}\,{\rm{fiber}}+ \% \,{\rm{ash}})$$

Fatty acid, amino acid, and mineral analysis were carried out following AOAC^8^. Calcium, magnesium, chromium, manganese, iron, chromium analysis was performed in Absorption Spectrophotometer (Model 280 FS- Make Agilent Technology). Phosphorus content was estimated using UV-Vis Spectrophotometer (Model - Agilent Carry-60). Amino acids were analyzed using HPLC (Model - Shimadzu LC-20 AD/T)). Fatty acids were analyzed through gas chromatography (Model - Shimadzu 2010 Plus).

### Sensory evaluation test

The sensory evaluation test was carried out using nine-point hedonic scale^9^ for comparing aroma, taste, and muscle texture between Hooghly hilsa and Padma hilsa. A panel of 50 evaluators was assigned for this test. Three types of cooked items, i.e., fried hilsa, steamed hilsa and hilsa curry from each portion (anterior, middle and posterior) of the fish sample were prepared, and all the three items from each portion of fish were given thrice to each evaluator. Briefly, the method of preparation for fried hilsa, dressed fish was marinated for 10 minutes with salt and turmeric powder followed by frying using mustard oil as per the traditional hilsa cuisine in West Bengal. For the preparation of steamed hilsa, mustard paste, turmeric powder, cumin powder, slit green chili, salt, and mustard oil was applied over the dressed fish and mixed thoroughly and marinated for 10 minutes followed by steam cooking. For the preparation of hilsa curry, dressed fish was marinated with turmeric powder, chili powder, and salt. Mustard seed, poppy seed, and green chili were minced together, and the paste along with cumin powder, turmeric powder and chili powder was transferred to pan mildly pre-fried in mustard oil then water was added and boiled as in traditional Bengali cuisine. After a few minutes of boiling, marinated fish was added for making curry. The sensory evaluation was based on a total of nine points from ‘like extremely’ to ‘dislike extremely’ and involved four parameters viz., smell, taste, texture of muscle, and overall grading.

### Statistical analysis

The means and standard errors were compared using standard statistical procedures. One-way ANOVA was used to determine the significant difference of nutritional composition among different size groups of hilsa. For comparative nutritional composition and sensory evaluation of Hoogly and Padma adult hilsa t-test was performed. All analysis was performed using IBM SPSS version 21.0 (SPSS Inc., Chicago, IL, USA). If the main effect was significant, the ANOVA was followed by Tukey's test at P < 0.05 and P < 0.01 level of significance.

## Results and Discussion

### Nutritional composition of different size groups of Hilsa

Body composition of fish depends on the feeding habit of that particular fish^10^. Hilsa is an omnivore and feeds on both phytoplankton and zooplankton. Nutrient content, mainly protein, lipid, amino acid, and fatty acid of the fish muscle depends on the feeding habit, type of feed, and abundance of feed^11,12^. Feed quality and feeding rate can also affect the body composition of fish, particularly crude protein, lipid, and moisture content^13^. Higher lipid and fatty acids mainly DHA content in fish muscle can be attributed to the high level of lipid and DHA in their feed^14,15^.

#### Proximate composition

Proximate composition of different size group of hilsa (Table 2) revealed that moisture and crude protein contents were significantly (P < 0.01) higher in groups I and II as compared to the other groups and the values gradually decreased as the weight of fish increased. In contrast, total lipid (%) was significantly (P < 0.01) lower in group I, and it significantly (P < 0.01) increased as the weight of the fish increased. Lipid content (%) was lowest in group I (5.68 ± 0.49) and highest in group VII (26.87 ± 0.43). Ash content (%) also significantly (P < 0.01) differed among the various size groups of fish. It was lowest in group I (13.25 ± 0.13 %) and increased gradually with larger size groups. Nitrogen free extract (carbohydrate) was significantly (P < 0.01) higher in smaller size groups as compared to larger size groups. Carbohydrate content (%) was maximum in group I (19.18 ± 1.62) and decreased as the weight of fish increased.

**Table 2:** Proximate composition (mean ± SE) of different size groups of hilsa collected from Hooghly river system.

Proximate Composition	Group I (n = 50)	Group II (n = 50)	Group III (n = 50)	Group IV (n = 50)	Group V (n = 50)	Group VI (n = 50)	Group VII (n = 50)
Moisture (%)**	75.06^d^ ± 0.45	72.10^d^ ± 1.29	52.43^c^ ± 2.33	50.16^bc^ ± 0.73	41.29^a^ ± 0.61	42.83^ab^ ± 0.95	41.02^a^ ± 0.45
Crude protein (%)**	61.78^c^ ± 1.15	60.38^c^ ± 0.86	52.08^b^ ± 0.09	50.17^ab^ ± 0.21	51.97^b^ ± 0.83	50.24^ab^ ± 0.72	49.51^a^ ± 0.3
Lipid (%)**	5.68^a^ ± 0.49	11.55^b^ ± 1.96	20.25^c^ ± 0.8	24.18^d^ ± 0.71	25.04^d^ ± 1.08	26.13^d^ ± 1.58	26.87^d^ ± 0.43
Ash (%)**	13.25^a^ ± 0.13	15.75^b^ ± 0.41	17.54^bc^ ± 0.1	19.28^cd^ ± 1.17	20.76^d^ ± 1.4	21.02^d^ ± 0.6	21.47^d^ ± 0.16
Nitrogen free extract (%)**	19.18^d^ ± 1.62	12.16^c^ ± 1.78	9.91^c^ ± 0.63	6.03^b^ ± 0.8	1.81^a^ ± 0.77	2.15^a^ ± 0.8	1.63^a^ ± 0.35

**P < 0.01 Means followed by different superscripts, differ significantly from each other.

In the present study, crude protein and carbohydrate contents of small size groups of hilsa were higher, indicating higher nutrient requirement due to the higher metabolic activity of small fish^16^. When dietary energy exceeds the energy requirement as normally happens in adult fishes when metabolic activity comes down, energy is stored as lipid in the body as was observed in the present study in large size groups of fish, where lipid content increased. The lipid content inversely related to the moisture content^17^. In Atlantic salmon, the body lipid decreased as they migrated to upstream rivers^2^. In hilsa, fat content varies during migration. The fat content increases as the fish enter brackish water from the marine environment and thereafter gradually decrease when they move into freshwater areas. Hilsa accumulates fat in the marine and brackishwater environments and migrates to freshwater upstream and spends their fat for spawning^18^. This may be the reason for higher lipid content in hilsa collected from the Hooghly estuary than those collected from the upstream stretch of Padma river. After upward migration and spawning, the lipid content of the fish decreased^18^. The present study revealed a gradual increase in lipid content in proportion to the size of the fish as it grew. In an earlier report^19^ hilsa (848.9 ± 86.8) from the Bay of Bengal were reported to contain 50.31 % crude protein, which corroborated with the present findings. In contrast to the present finding, higher lipid level was reported^19^ even in similar size groups of hilsa. In general, small size fish having higher metabolic activity meet their energy requirement mainly from carbohydrates (the principal energy source), which is corroborated by the presence of higher carbohydrate content in small size groups (<5 g). Migratory fishes like trout utilize carbohydrate for energy and spare protein for tissue building as protein is only utilized for energy if the available energy from other sources (lipid and carbohydrates) is insufficient^20^. However, in the present study, carbohydrate levels did not vary significantly in the different size groups (10 g, 200 g, 500 g, and 800 g) as hilsa do not depend on carbohydrate to draw energy for migration^6^.

#### Mineral composition

Among minerals, calcium, phosphorus, magnesium, zinc, manganese, chromium, and iron content were significantly (P < 0.01) higher in group I compared to other groups (Table 3). Calcium and phosphorous contents (%) varied between 0.37 to 0.99 and 0.32 to 0.65, respectively. No significant difference (P > 0.05) in Ca:P ratio in body composition of different size groups was observed, and it ranged from 1.01 to 1.52. Magnesium, chromium, manganese, zinc, and iron content (ppm) ranged between 229.25–394.40, 0.29–0.66, 1.75–6.87, 9.85–38.79 and 18.00–62.38, respectively in different size groups.

**Table 3:** Mineral content (mean ± SE) of different size groups of hilsa collected from Hooghly river system.

Mineral content	Group I	Group II	Group III	Group IV	Group V	Group VI	Group VII
Calcium**(g%)	0.99^d^ ± 0.01	0.42^ab^ ± 0.01	0.54^bc^ ± 0.04	0.38^a^ ± 0.02	0.37^a^ ± 0.02	0.43^ab^ ± 0.04	0.6^c^ ± 0.06
Phosphorus**(g%)	0.65^e^ ± 0.02	0.42^cd^ ± 0.02	0.36^ab^ ± 0.01	0.34^ab^ ± 0.004	0.32^a^ ± 0.02	0.38^bc^ ± 0.02	0.47^d^ ± 0.02
Magnesium **(ppm)	394.4^e^ ± 2.90	296.85^d^ ± 1.95	281.1^c^ ± 2.40	247.95^b^ ± 1.05	229.25^a^ ± 0.75	231.7^a^ ± 0.40	284.1^c^ ± 2.30
Chromium **(ppm)	0.66^g^ ± 0.01	0.41^c^ ± 0.003	0.51^f^ ± 0.004	0.29^a^ ± 0.001	0.50^e^ ± 0.002	0.33^b^ ± 0.001	0.47^d^ ± 0.004
Manganese** (ppm)	6.87^g^ ± 0.05	3.58^e^ ± 0.02	3.06^c^ ± 0.03	3.82^f^ ± 0.02	1.75^a^ ± 0.01	2.05^b^ ± 0.004	3.27^d^ ± 0.03
Zinc**(ppm)	38.79^g^ ± 0.29	18.13^f^ ± 0.11	9.85^a^ ± 0.09	10.80^b^± 0.04	14.29^d^ ± 0.05	12.71^c^ ± 0.02	15.64^e^ ± 0.13
Iron **(ppm)	62.38^g^ ± 0.45	20.78^c^ ± 0.14	18.96^b^ ± 0.17	18.00^a^ ± 0.07	38.28^e^ ± 0.13	46.43^f^ ± 0.02	26.94^d^ ± 0.22

**P < 0.01 Means followed by different superscripts, differ significantly from each other.

Mineral content was higher in smaller size groups compared to larger groups indicating that hilsa fry requires a higher amount of minerals in their diet. Ash content of all size groups in the present study was higher than those reported by earlier on the same species^19^. Higher ash content could be due to the fact that in the present study samples that were analyzed contained bones/spines. Calcium, phosphorus, iron, and zinc content in smaller size groups of hilsa were higher than those of other fishes^21^. The study revealed higher levels of calcium and lower levels of phosphorus in all size groups of hilsa than the value reported earlier for the fish from riverine and marine sources^18^. The recommended range of calcium to phosphorus ratio in fish is 1:1 to 1:1.7^22^, but calcium content recorded in this study was higher than phosphorus, which can be attributed to higher bone content in the selected samples.

#### Amino acid composition

Among essential amino acids (EAA), arginine, methionine, and threonine content were significantly (P < 0.01) higher in muscles of group I compared to other groups (Table 4). Leucine and isoleucine contents were significantly (P < 0.01) higher in groups IV and VII as compared to the other size groups. Valine, phenylalanine, and histidine contents were variable in the different groups. Among non-essential amino acid (NEAA) aspartic acid and glutamic acid content were significantly (P < 0.01) higher in groups V and VI, and glycine content was significantly (P < 0.05) higher in muscles of group I compared to the other groups (Table 4).

**Table 4:** Amino acid composition (mean ±SE) of hilsa of different size groups collected from Hooghly river system.

Amino acid	Group I	Group II	Group III	Group IV	Group V	Group VI	Group VII
Threonine**	4.09^b^ ± 0.09	4.66^b^ ± 0.01	3.12^a^ ± 0.05	2.43^a^ ± 0.07	4.67^b^ ± 0.51	4.7^b^ ± 0.12	3.12^a^ ± 0.05
Valine**	3.69^b^ ± 0.04	4.67^c^ ± 0.005	3.59^b^ ± 0.06	2.80^a^ ± 0.14	6.06^e^ ± 0.29	5.38^d^ ± 0.03	3.65^b^ ± 0.04
Methionine**	3.22^c^ ± 0.17	ND	0.10^ab^ ± 0.03	ND	ND	0.26^b^ ± 0.02	0.01^a^ ± 0.00
Isoleucine**	4.52 ± 0.31b	4.99^c^ ± 0.24	8.53^d^ ± 0.09	11.98^f^ ± 0.52	3.08^a^ ± 0.40	3.79^ab^ ± 0.05	10.21^e^ ± 0.09
Leucine**	12.86^a^ ± 0.34	14.69^a^ ± 0.37	22.43^b^ ± 0.24	32.22^c^ ± 1.39	13.00^a^ ± 0.86	11.49^a^ ± 0.36	30.52^c^ ± 2.84
Phenyl alanine**	5.07^d^ ± 0.12	4.78^cd^ ± 0.14	2.16^a^ ± 0.04	3.84^bc^ ± 0.61	3.82^bc^ ± 0.38	3.39^b^ ± 0.07	3.14^b^ ± 0.16
Histidine*	4.11^bc^ ± 0.14	2.71^a^ ± 0.005	4.4^d^ ± 0.94	3.02^ab^ ± 0.01	2.81^ab^ ± 0.28	2.33^ab^ ± 0.03	2.11^a^ ± 0.01
Lysine*	7.14^bc^ ± 0.52	5.90^ab^ ± 0.23	4.68^a^ ± 0.14	6.86^bc^ ± 0.07	6.18^bc^ ± 0.89	7.45^c^ ± 0.22	6.67^bc^ ± 0.21
Arginine**	16.53^f^ ± 0.21	10.95^e^ ± 0.82	4.13^bc^ ± 0.41	6.29^d^ ± 0.17	1.85^a^ ± 0.02	2.95^ab^ ± 0.32	4.97^cd^ ± 1.05
∑EAA**	61.21^c^ ± 0.020	53.34^b^ ± 1.54	53.12^b^ ± 1.43	69.40^d^ ± 1.03	41.45^a^ ± 3.62	41.73^a^ ± 1.02	64.37^cd^ ± 1.26
Aspartic acid**	4.44^ab^ ± 0.17	6.09^c^ ± 0.20	7.89^d^ ± 0.69	3.4^a^ ± 0.02	10.25^e^ ± 0.53	11.11^e^ ± 0.01	4.74^b^ ± 0.05
Serine**	2.07^ab^ ± 0.02	2.51^bc^ ± 6.90	2.41^bc^ ± 0.36	1.12^a^ ± 0.01	4.08^d^ ± 0.68	3.36^cd^ ± 0.06	1.60^ab^ ± 0.02
Glutamic acid**	3.11^a^ ± 0.25	6.85^b^ ± 0.03	10.54^c^ ± 0.47	4.22^ab^ ± 0.38	12.17^c^ ± 2.51	12.60^c^ ± 0.19	5.67^ab^ ± 0.18
Proline*	1.08^a^ ± 0.35	2.65^ab^ ± 0.57	0.56^a^ ± 0.23	3.88^b^ ± 1.49	0.66^a^ ± 0.19	1.14^a^ ± 0.68	4.19^b^ ± 0.11
Glycine*	23.04^c^ ± 0.20	5.14^c^ ± 0.65	7.62^a^ ± 0.53	12.23^b^ ± 0.01	13.13^b^ ± 0.59	12.44^b^ ± 0.07	11.31^b^ ± 0.47
Alanine**	3.36^ab^ ± 0.18	1.00^ab^ ± 0.11	14.55^c^ ± 0.45	2.48^a^ ± 0.17	14.56^c^ ± 1.87	14.01^c^ ± 0.52	5.46^b^ ± 0.35
Cysteine*	ND	2.08^ab^ ± 0.57	ND	ND	0.37^b^ ± 0.04	0.35^b^ ± 0.06	0.12^a^ ± 0.01
Tyrosine**	0.69^ab^ ± 0.06	0.89^bc^ ± 0.11	0.83^ab^ ± 0.15	0.99^bc^ ± 0.16	1.34^d^ ± 0.05	1.3^cd^ ± 0.06	0.54^a^ ± 0.06
∑NEAA**	37.78^ab^ ± 0.02	45.41^c^± 1.79	44.38^d^ ± 0.92	28.30^a^ ± 1.13	56.51^e^ ± 3.65	56.28^e^ ± 1.02	33.62^b^ ± 1.24

**P < 0.01, *P < 0.05, ND-not detected, Means followed by different superscripts differ significantly from each other.

Essential amino acids levels were higher than total non-essential amino acids in hilsa muscle indicated high quality of muscle protein^23^. Apart from essential and non-essential amino acids, a new group of amino acids, i.e., functional amino acids play an important role in regulating the metabolic pathways in the body. They are mainly arginine, cysteine, leucine, methionine, tryptophan, tyrosine, aspartate, glutamic acid, glycine, and proline^4^. Leucine and glycine content of hilsa muscle were higher compared to salmon^24^ and small indigenous fishes^2^. All EAAs and NEAAs content of *Tenualosa ilisha* reported earlier were lower compared to our findings^4^. Among all amino acids, the leucine content was highest followed by glycine, alanine, and glutamic acid in all size groups of hilsa.

Leucine has a key role in muscle protein synthesis and also plays an important role in stress conditions^4^. The higher functional amino acid content in hilsa fry and juveniles revealed higher requirement of glycine, arginine, methionine in their diet.

#### Fatty acid composition

Fatty acid analysis (Table 5) revealed that total saturated fatty acids (SFA) and mono-unsaturated fatty acids (MUFA) were significantly (P < 0.01) lower in group I as compared to the other groups. In contrast, total PUFA content was significantly (P < 0.05) higher in group I. Among SFAs, C16 palmitic acid (P < 0.05), C18 stearic acid (P < 0.01) and C14 myristic acid (P < 0.01) were significantly lower in group I. Among MUFAs, oleic acid and palmitoleic acid were lower (P < 0.05) in group I as compared to the fish belonging to larger size groups. Among the PUFAs, arachidonic acid, DHA was significantly (P < 0.01) higher in group I whereas, EPA was significantly (P < 0.01) higher in larger size groups. Both n-3 and n-6 PUFAs were significantly (P < 0.01) higher in group I. Ratio of n3 to n6 fatty acids was significantly (P < 0.01) higher in group VI followed by group V and lowest in group VII.

**Table 5:** Fatty acid composition (mean ± SE) of different size groups of hilsa collected from Hooghly river system.

Fatty acid	Group I	Group II	Group III	Group IV	Group V	Group VI	Group VII
C14**	1.82^a^ ± 0.23	7.76^b^ ± 0.21	7.32^cd^ ± 0.01	7.28^cd^ ± 0.37	7.00^c^ ± 0.13	8.51^e^ ± 0.21	6.00^b^ ± 0.06
C15	ND	0.18 ± 0.18	ND	ND	ND	ND	ND
C16*	15.92^a^ ± 1.45	25.49^b^ ± 4.14	26.04^b^ ± 0.34	24.78^b^ ± 0.56	24.09^b^ ± 0.18	26.38^b^ ± 0.57	24.34^b^ ± 0.19
C17	0	0.11 ± 0.11	ND	ND	ND	ND	ND
C18**	7.01^a^ ± 0.83	9.45^ab^ ± 2.85	13.26^bc^ ± 0.80	15.86^c^ ± 0.56	14.04^bc^ ± 2.03	10.58^ab^ ± 1.04	24.43^d^ ± 0.61
∑SFA**	24.75^a^ ± 0.85	42.99^b^ ± 1.22	46.62^bc^ ± 1.15	47.92^c^ ± 0.37	45.13^bc^ ± 1.96	45.47^bc^ ± 0.25	54.77^d^ ± 0.48
C16:1**	3.77^a^ ± 0.36	13.34^c^ ± 2.00	12.90^c^ ± 0.04	12.18^c^ ± 0.27	13.26^c^ ± 0.06	13.23^c^ ± 0.43	7.17^b^ ± 0.18
C17:1	ND	0.02 ± 0.02	ND	ND	ND	ND	ND
C18:1*	5.96^a^ ± 0.50	12.865^b^ ± 2.405	14.45^b^ ± 0.29	13.70^b^ ± 0.23	18.52^b^ ± 3.49	12.83^b^ ± 0.53	13.82^b^ ± 0.26
C20:1	ND	0.345 ± 0.345	ND	ND	ND	ND	1.56 ± 0.02
∑MUFA**	9.73^a^ ± 0.16	26.57^b^ ± 4.04	27.35^b^ ± 0.24	25.88^b^ ± 0.51	31.78^b^ ± 3.55	26.06^b^ ± 0.96	22.55^b^ ± 0.45
C18:2*	1.72^a^ ± 0.04	2.49^c^ ± 0.29	1.94^ab^ ± 0.04	2.52^c^ ± 0.12	2.26^bc^ ± 0.05	2.51^c^ ± 0.20	1.94^ab^ ± 0.01
C18:3	0.91 ± 0.06	0.77 ± 0.35	ND	ND	ND	ND	ND
C20:2	ND	ND	ND	ND	ND	ND	1.07 ± 0.01
C20:3	ND	0.145 ± 0.145	ND	ND	ND	ND	ND
C20:4**	6.67^d^ ± 0.01	2.02^ab^ ± 0.02	2.16^bc^ ± 0.05	1.93^a^ ± 0.05	ND	ND	2.29^c^ ± 0.03
C20:5*	9.27^a^ ± 0.17	15.92^bc^ ± 3.51	15.2^bc^ ± 0.51	15.39^bc^ ± 0.51	14.78^bc^ ± 1.11	18.45^c^ ± 0.35	12.18^ab^ ± 0.01
C22:6**	46.98^d^ ± 0.66	8.80^c^ ± 1.29	6.75^ab^ ± 0.30	6.34^ab^ ± 0.14	6.06^ab^ ± 0.41	7.53^bc^ ± 0.14	5.25^a^ ± 0.01
∑PUFA**	65.55^c^ ± 0.71	30.15^b^ ± 4.9	26.05^ab^ ± 0.91	26.18^ab^ ± 0.81	23.10^ab^ ± 1.57	28.49^ab^ ± 0.69	22.73^a^ ± 0.04
n-3**	57.15^c^ ± 0.76	25.49^b^ ± 4.45	21.95^ab^ ± 0.81	21.72^ab^ ± 0.64	20.84^ab^ ± 1.52	25.98^b^ ± 0.49	17.43^a^ ± 0.01
n-6**	8.39^c^ ± 0.06	4.66^b^ ± 0.455	4.09^ab^ ± 0.09	4.44^ab^ ± 0.17	2.26^ab^ ± 0.05	2.51^e^ ± 0.20	4.22^a^ ± 0.02
n-3/n-6**	6.81^c^ ± 0.14	5.43^b^ ± 0.42	5.36^b^ ± 0.07	4.89^b^ ± 0.05	9.21^d^ ± 0.47	10.4^e^ ± 0.63	4.13^a^ ± 0.02

**P < 0.01, * P < 0.05, ND-not detected, Means followed by different superscripts in a row differ significantly from each other.

Fishes contain good quality fatty acids such as DHA and EPA, which help in preventing coronary heart diseases in humans^25^. Hilsa used in the present study had 22.73 to 65.55 % PUFA which was higher than the earlier report in marine hilsa (11.41%) and freshwater hilsa (26.87%)^21^. Among PUFA, the proportions of DHA and EPA were high in all the size groups. The DHA was significantly (P < 0.01) higher in juvenile hilsa, and its concentration significantly reduced with age. However, total SFA and MUFA concentration increased proportionately with an increase in body weight. The result indicated that the requirement of DHA is higher for early age groups, whereas saturated and mono-unsaturated fatty acid requirement increases with the advancement of age. The values of total SFA and MUFA of the large size group (> 800 g) of hilsa in the present study are in line with earlier report^18^. The SFA was significantly (P < 0.01) higher in large fish (>800 g), whereas PUFA was significantly (P < 0.01) lower. MUFA was similar in all the groups above 5 g. Similar to the present findings, lower PUFA content in large hilsa compared to juvenile hilsa has also been reported earlier^6^. This variation in PUFA is mainly due to changes in feeding habit^26^ as hilsa in their early stages prefer copepods, rich in PUFA, unlike adult hilsa, which prefer diatoms^27^. Although n-3 and n-6 PUFA content were lower in large size fish, n-3/ n-6 PUFA was higher as reported earlier^28^ due to very less content of n-6 PUFA in large size fish. In the present study, n-3 to n-6 PUFA ratio was found to be within a range of 4.31 to 10.40, which corroborates earlier reports^6^. The DHA was the most dominant PUFA followed by EPA and arachidonic acid in juvenile hilsa of below 5 g, which is similar to that reported earlier in the clupeid fish *Sardinella lemuru*^29^. DHA content of hilsa of <100 g size was similar to the DHA content reported for salmon of the same size group^12^.

### Comparison of body composition and taste of hilsa from Hooghly and padma rivers

**Figure 2:**
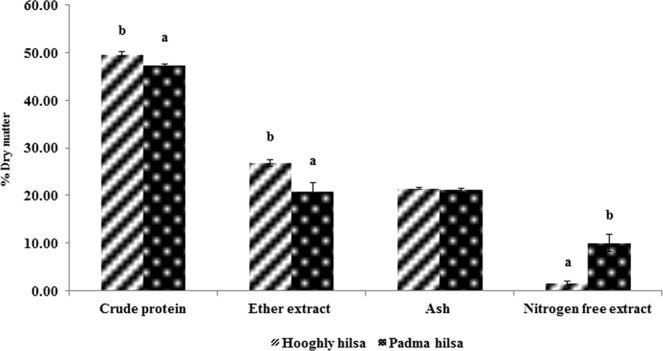
Comparison of proximate composition (mean ± SD) of hilsa collected from Hooghly and Padma river. Figures with different superscripts indicate significant differences **P < 0.01.

Comparison of proximate composition (Fig. 2) between hilsa from Hooghly (837.50 ± 8.62 g/ 402.13 ± 3.60 mm size) and Padma (832.27 ± 7.87 g/401.88 ± 2.64 mm size) collected during the same season revealed that Hooghly stock contained significantly (P < 0.01) higher crude protein and fat but Padma stock contained significantly(P < 0.01) higher amount of carbohydrate. The Hooghly stock contained significantly (P < 0.05) higher amount of calcium and phosphorus compared to the Padma stock. Magnesium, manganese, chromium and zinc content of Hooghly stock were also higher (P < 0.01) whereas, iron content was significantly (P < 0.01) lower than the Padma stock (Table 6).

**Table 6:** Comparison of Mineral composition (mean±SE) of adult hilsa (>800 g) from Hooghly and Padma river systems.

Mineral	Specimens	
	Hooghly hilsa	Padma hilsa
Calcium (g%)*	0.60^b^ ± 0.06	0.21^a^ ± 0.03
Phosphorus (g%)*	0.47^b^ ± 0.02	0.23^a^ ± 0.02
Magnesium (ppm)**	284.1^b^ ± 2.30	194.2^a^ ± 1.70
Chromium (ppm)**	0.47^b^ ± 0.004	0.37^a^ ± 0.003
Iron (ppm)**	26.94^a^ ± 0.22	31.58^b^ ± 0.28
Manganese (ppm)**	3.27^b^ ± 0.03	0.95^a^ ± 0.01
Zinc (ppm)**	15.64^b^ ± 0.13	9.49^a^ ± 0.08

*P < 0.05, **P < 0.01, Means followed by different superscripts differ significantly from each other.

The amino acid content in the body of hilsa from Hooghly and Padma rivers (Table 7) revealed higher (P < 0.05) total EAA in the Hooghly stock and higher (P < 0.01) total NEAA in the Padma stock. Among EAA, isoleucine content was significantly (P < 0.01) higher in the Hooghly stock, and threonine and valine were significantly (P < 0.01) higher in the Padma stock. Among NEAA, aspartic acid, serine, glutamic acid, alanine (P < 0.01), cysteine, and tyrosine (P < 0.05) contents were significantly higher in the Padma stock, whereas, glycine (P < 0.05) and proline (P < 0.01) contents were significantly higher in hilsa from the Hooghly estuary.

**Table 7:** Comparison of amino acid composition (mean±SE) of adult hilsa (>800 g) from Hooghly and Padma river systems.

Amino acid	Specimens	
	Hooghly hilsa	Padma hilsa
Threonine**	3.12^a^ ± 0.05	4.73^b^ ± 0.03
Valine**	3.65^a^ ± 0.04	5.25^b^ ± 0.09
Methionine	0.01 ± 0.00	0.00
Isoleucine**	10.21^b^ ± 0.09	2.85^a^ ± 0.62
Leucine*	30.52^b^ ± 2.84	10.65^a^ ± 0.325
Phenylalanine	3.14 ± 0.16	2.81 ± 0.11
Histidine	2.11 ± 0.01	1.96 ± 0.05
Lysine	6.67 ± 0.21	4.70 ± 1.01
Arginine	4.97 ± 1.05	0.93 ± 0.04
Total EAA**	64.40^b^ ± 1.26	33.88^a^ ± 1.19
Aspartic acid**	4.74^a^ ± 0.05	12.81^b^ ± 0.03
Serine**	1.60^a^ ± 0.02	4.08^b^ ± 0.11
Glutamic acid**	5.67^a^ ± 0.18	17.70^b^ ± 0.46
Proline**	4.19^b^ ± 0.11	0.32^a^ ± 0.04
Glycine*	11.31^b^ ± 0.47	8.09^a^ ± 0.40
Alanine**	5.46^a^ ± 0.35	19.83^b^ ± 0.05
Cysteine*	0.12^a^ ± 0.01	0.33^b^ ± 0.03
Tyrosine*	0.54^a^ ± 0.06	1.00^b^ ± 0.08
Total NEAA**	33.62^a^ ± 1.24	64.16^b^ ± 1.19

*P < 0.05, **P < 0.01, Means followed by different superscripts in a row differ significantly from each other.

The fatty acid content in the body of hilsa (Table 8) revealed that stearic acid and total SFA was significantly (P < 0.01) higher in the Hooghly stock whereas, total MUFA was significantly (P < 0.01) higher in the Padma stock. Palmitoleic acid and cis-oleic acid were significantly (P < 0.01) higher in the Padma stock, whereas, arachidonic acid, EPA, and DHA were slightly higher (P > 0.05) in the Hooghly stock. No significant difference in total PUFA content was found between the Hooghly and Padma stocks. Total n-6 fatty acid content was found to be higher (P < 0.05) in the Hooghly stock as compared to the Padma stock. But no significant difference was found in total n-3 fatty acid content between the Hooghly and Padma stocks. The ratio of n3 to n6 fatty acid was significantly (P < 0.01) higher in the Padma stock (3.82) as compared to that from the Hooghly (3.30).

**Table 8:** Comparison of fatty acid composition (mean ± SE) of adult hilsa (>800 g) from Hooghly and Padma river systems.

Fatty acid	Specimens	
	Hooghly hilsa	Padma hilsa
C14	6.00 ± 0.06	6.14 ± 0.10
C16	24.34 ± 0.19	23.88 ± 0.09
C18**	24.43^b^ ± 0.61	15.33^a^ ± 0.20
∑SFA**	54.77^b^±0.48	45.35^a^ ± 0.20
C16:1**	7.17^a^ ± 0.18	11.86^b^ ± 0.20
C18:1**	13.82^a^ ± 0.26	20.76^b^ ± 0.55
C20:1**	1.56^b^ ± 0.02	1.25^a^ ± 0.02
C22:1**	0	0.14 ± 0.11
∑MUFA**	22.54^a^±0.45	33.86^b^ ± 0.76
C18:2**	1.94^b^ ± 0.01	1.79^a^ ± 0.00
C20:2**	1.06^b^ ± 0.01	0.87^a^ ± 0.01
C20:4	2.29 ± 0.03	1.63 ± 0.20
C20:5	12.18 ± 0.005	11.78 ± 0.10
C22:6	5.25 ± 0.01	4.59 ± 0.74
∑PUFA	22.71 ± 0.04	20.66 ± 0.84
n-3	17.42 ± 0.02	16.37 ± 0.64
n-6*	5.28^b^ ± 0.02	4.29^a^ ± 0.20
n-3/n-6**	3.30^a^ ± 0.01	3.82^b^ ± 0.03

*P < 0.05, **P < 0.01, Means followed by different superscripts in a row differ significantly from each other.

Sensory evaluation result (Table 9) revealed that aroma, taste, and muscle texture of the Padma stock were significantly (P < 0.05) superior to hilsa from the Hooghly river. There was 5.88, 4.75 and 5.48% higher score for smell, taste, and texture of muscle in Padma hilsa compared to the muscle of Hooghly hilsa.

**Table 9:** Comparative sensory evaluation of adult hilsa collected from the Padma and Hooghly river using nine-point hedonic scale.

Specimens		
Parameter	Hooghly hilsa	Padma hilsa
Smell*	6.46^a^ ± 0.11	6.84^b^ ± 0.10
Taste*	6.53^a^ ± 0.11	6.84^b^ ± 0.12
Texture of muscle*	6.21^a^ ± 0.12	6.55^b^ ± 0.12
Overall grading*	6.66^a^ ± 0.11	7.00^b^ ± 0.11

*P < 0.05, Means followed by different superscripts in a row differ significantly from each other.

Hilsa is considered as one of the tastiest fishes due to its unique texture and flavor. In general, the taste and flavor of fishes depend on their food and feeding behavior^30^. The flavor depends on the presence of flavor-producing compounds, which greatly vary from species to species and the biological condition of the fish. Taste and flavor are mainly formed by the associated effect of glutamic acid and nucleotide, along with sodium and chloride ions^31^. The exclusive taste of hilsa has often been credited to the presence of fatty acids like stearic acid, oleic acid, linoleic, linolenic, arachidonic, EPA, and DHA^2,6,21^. There is a widely accepted perception that hilsa from Padma river system is tastier than that of hilsa from Hooghly, but the correlation between the taste and nutrient contents of hilsa from the two riverine systems was not evaluated scientifically so far. Lower (P < 0.01) lipid content in hilsa of Padma compared to that from Hooghly might be due to higher utilization of body lipid during upstream migration, which makes the fish more soft and lean. During upward migration, saturated fatty acids are transformed first into mono- and then into polyunsaturated fatty acids. Upstream migratory fish or anadromous fish like hilsa, Atlantic salmon, Chum salmon, and Sockeye salmon generally convert saturated fatty acids into mono or polyunsaturated fatty acids during their upstream migration^17,32^. Higher MUFA, especially oleic acid in hilsa from the Padma, is one of the factors partly responsible for better texture and characteristic flavor in the muscles. The ratio of n3 to n6 fatty acid also influenced the flavor of fish, as found in saithe, *Pollachius virens*, where higher n3 to n6 fatty acid, was reported to have better flavor compared to fish with lower n3 to n6 fatty acid ratio^33^. It has been reported that amino acids such as glycine, glutamic acid, and alanine are taste and flavor-enhancing factors in food and have a role in a pleasant sweet taste^34^. Higher content of glutamic acid, alanine and aspartic acid which are responsible for enhancing flavor along with higher oleic acid, palmitoleic acid, and higher n3 to n6 fatty acid ratio might have attributed to slightly better muscle texture, and flavor of Padma hilsa compared to Hooghly hilsa. Type of food available and feeding habits in different locations or ion transport mechanism or osmoregulation during the shift in the migratory pattern of fishes can also contribute to differences in texture of muscles which need further investigation. Though the hilsa from the Padma were found to have superior taste in the present study, the higher protein, EAA, mineral and highly unsaturated fatty acid content in hilsa from Hooghly river estuary indicated their better nutritional quality compared to those from the Padma river.

## Conclusion

Nutritional composition of different size groups of hilsa collected from different habitats gives insight into the nutrient requirement of hilsa at different stages of growth, which would help in the formulation of balanced feeds for the fish in attempts to culture them. Moreover, the taste and flavor of fish are greatly governed by the living conditions or food and feeding habit of the fish. The nutritional profile of hilsa from different locations throws light on the various taste enhancing amino acids or fatty acid compounds in the fish, which could be attributed to its flavor. The findings open the possibility of incorporating these components into the artificial feed to enhance the taste and acceptability of hilsa.

## Data Availability

The datasets generated during and/or analysed during the current study are available from the corresponding author on reasonable request.
